# MASCP gator: an overview of the Arabidopsis proteomic aggregation portal

**DOI:** 10.3389/fpls.2013.00411

**Published:** 2013-10-23

**Authors:** Gregory W. Mann, Paul C. Calley, Hiren J. Joshi, Joshua L. Heazlewood

**Affiliations:** ^1^Joint BioEnergy Institute and Physical Biosciences Division, Lawrence Berkeley National LaboratoryBerkeley, CA, USA; ^2^Copenhagen Center for Glycomics, Institute for Cellular and Molecular Medicine, University of CopenhagenCopenhagen, Denmark

**Keywords:** proteomics, Arabidopsis, mass spectrometry, database, protein modifications, single nucleotide polymorphisms

## Abstract

A key challenge in the area of bioinformatics in the coming decades is the ability to manage the wealth of information that is being generated from the variety of high throughput methodologies currently being undertaken in laboratories across the world. While these approaches have made available large volumes of data to the research community, less attention has been given to the problem of how to intuitively present the data to enable greater biological insights. Recently, an attempt was made to tackle this problem in the area of Arabidopsis proteomics. The model plant has been the target of countless proteomics surveys producing an exhaustive array of data and online repositories. The MASCP Gator is an aggregation portal for proteomic data currently being produced by the community and unites a large collection of specialized resources to a single portal (http://gator.masc-proteomics.org/). Here we describe the latest additions, upgrades and features to this resource further expanding its role into protein modifications and genome sequence variations.

## Background

The utilization of mass spectrometry for the identification of proteins from plant samples has been widely applied for the past decade (Heazlewood, 2011). The technology has been widely applied in large scale proteomic surveys of the model plant Arabidopsis (Castellana et al., 2008; Baerenfaller et al., 2011). These studies in Arabidopsis have resulted in the production of large quantities of data which are subsequently available through a variety of online portals and resources (Weckwerth et al., 2008). The construction of these boutique databases can result in a fragmentation of knowledge in a given biological system. In an attempt to mitigate this problem, the proteomics subcommittee of the Multinational Arabidopsis Steering Committee (MASCP) developed a data aggregation portal (MASCP Gator) to display proteomic data to the community (Joshi et al., 2011).

The MASCP Gator portal was originally made available in 2011 (http://gator.masc-proteomics.org/), and during the intervening period, the tool has been extensively updated, improving functionality and usability. A considerable amount of data and information have been added and the core sequences have been updated to the latest release available from The Arabidopsis Information Resource (TAIR) (Lamesch et al., 2012). Many improvements to the user interface have been made, making the tool easier to use, and exposing more of the tool for use in scripting environments. This article describes updates to the portal highlighting new features and summarizing the current state of protein identifications in the model plant Arabidopsis.

## Implementation of the MASCP gator

The original concept of the MASCP Gator was to provide a visual representation of distributed proteomics data that is both comprehensive and simple to use. The idea to connect a collection of boutique biological databases through a series of web services was not a new concept (Wilkinson and Links, 2002). The MASCP Gator was originally implemented using HTML, JavaScript and scalable vector graphics with a server-side component for caching and historical tracking of data written in JavaScript and the Node.JS runtime environment (Joshi et al., 2011). Web services were provided from the data sources in JSON format responding to a query based upon a given Arabidopsis Gene Identifier (AGI). The source code is available online at http://gator.masc-proteomics.org/source/ as well as documentation, unit tests, and examples so that individuals can utilize the libraries developed for the aggregator.

The initial construction of the MASCP Gator (Joshi et al., 2011) comprised aggregated information from SUBA (Heazlewood et al., 2007), AtProteome (Baerenfaller et al., 2008), ProMEX (Hummel et al., 2007; Wienkoop et al., 2012), PhosPhAt (Heazlewood et al., 2008; Durek et al., 2010; Arsova and Schulze, 2012), PPDB (Sun et al., 2009), RIPP-DB (Nakagami et al., 2010), AT_CHLORO (Ferro et al., 2010; Bruley et al., 2012), and AtPeptide (Castellana et al., 2008) and included underlying protein information and descriptions from TAIR (release 9) (Swarbreck et al., 2008). Since the initial release of the MASCP Gator a number of updates and changes have been undertaken. These have included the recent evolution of the AtProteome resource to pep2pro which coincided with updates to their analysis pipeline and the addition of new data (Baerenfaller et al., 2011; Hirsch-Hoffmann et al., 2012). Upgrades to the underlying protein models and descriptions have also been undertaken to incorporate TAIR10 (Lamesch et al., 2012).

**Figure 1:**
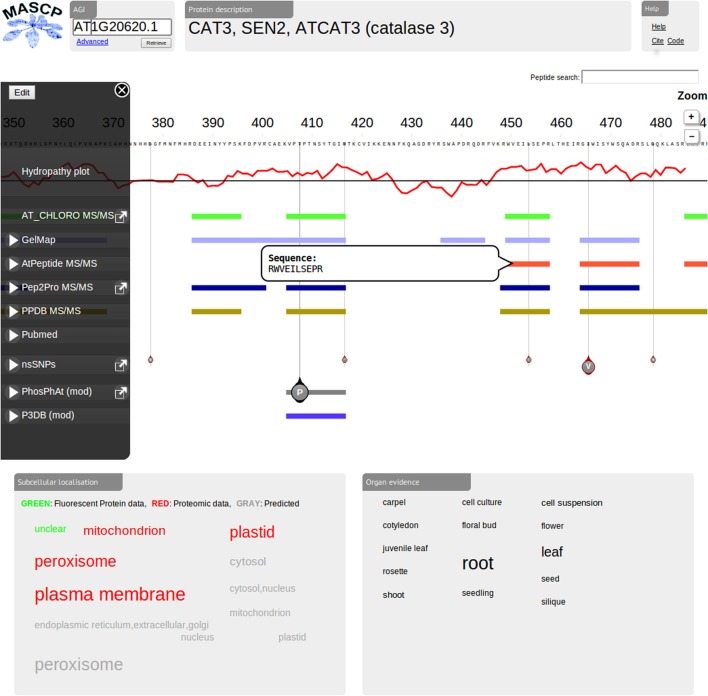
**Screenshot of the current release (version 2.0) of the MASCP Gator highlighting data for the AGI AT1G20620.1 (catalase 3).** A number of new features can be observed including the peptide search function (box top right), a peptide hover feature, enhanced track navigation feature (edit capabilities), the GelMap data track, the P^3^DB data track, the PubMed data track, and the 1001 Proteomes track (nsSNPs).

A screenshot of the current release interface for the MASCP Gator is shown in Figure 1. There are a number of new features and additions to the interface which will be outlined below.

## Recent updates to the MASCP gator

The MASCP Gator was designed in a modular manner to enable the incorporation of additional data sources as they are developed by the community. Moreover, the MASCP Gator's adoption of the NodeJS framework allows the rapid development of additional functionality including the introduction of static data sources.

A number of dynamic data sources have been added to the MASCP Gator since its initial development and release (Joshi et al., 2011). The Plant Protein Phosphorylation DataBase (P^3^DB) initially focused on providing protein phosphorylation data from a variety of plant species, with a major focus on *Brassica napus* (Gao et al., 2009). More recently the P^3^DB resource has begun housing experimental phosphorylation data from *Arabidopsis thaliana* as well as a range of other species including rice, Medicago, and soybean (Yao et al., 2012). Although much of the Arabidopsis data from P^3^DB overlaps considerably with existing phosphorylation resources already integrated with the MASCP Gator (e.g., PhosPhAt and RIPP-DB), redundancy is a useful mechanism to offset data stagnation from resource providers. The GelMap resource was also recently included and houses a range of two-dimensional gel electrophoresis images from plant extracts with mapped protein identifications from proteomic surveys (Rode et al., 2011). Its initial focus has been organelle proteomes from Arabidopsis, namely mitochondria and plastid, but this will likely expand as the community is encouraged to upload published data at this repository (Senkler and Braun, 2012). The inclusion of these resources brings the total number of peptides displayed by the MASCP Gator to over 10 million, which is comprised of around 220,000 unique peptides (Mann et al., 2013). The MASCP Gator displays information (experimental and non-experimental) for all 35,386 proteins currently available in TAIR10, which includes nearly 8000 proteins derived from alternate splicing models (designated as AGI code plus.2,.3,.4 and etc). Protein information will be displayed in the MASCP Gator at the alternate splice level if specified by a particular resource or publication. Where data only indicate information at the locus level, data is displayed for the representative gene model (generally the.1 version). In total, there is proteomic information for 24,811 Arabidopsis proteins covering over 70% of the potential proteome when compared to all proteins derived from alternate gene models (Table 1). The majority of the nearly 10,600 proteins with no experimental information mainly consist of alternate gene models (data not shown). A cursory examination of peptide coverage of the 24,811 proteins with experimental information shows that over 50% of the proteins have greater than 20% coverage (Figure S1). This simple analysis highlights the utility of data aggregation as these numbers suggest very good experimental evidence for nearly 12,500 Arabidopsis proteins.

**Table 1:** **Summary of proteomic derived information (August 2013) currently aggregated and displayed by the MASCP Gator**.

**Resource**	**Peptides**	**Proteins**
SUBA	N/A	9319^1^
pep2pro	6,178,840	21,451
PPDB	1,787,614	6905
ProMEX	695	248
AtPeptide	2,520,272	16,769
PhosPhAt	61,795	5486
AT_CHLORO	122,488	1263
RIPP-DB	5562	2244
P^3^DB	7575	3851
GelMap	21588	986
PubMed	21,774	2867

1 *The protein value for SUBA corresponds to proteins with experimental localization data (MS or FP)*.

A collection of static data related to protein features in Arabidopsis has also been included in the MASCP Gator. These include the addition of a PubMed track to capture published proteomics data not currently contained in online repositories (Roberts, 2001). The original publication of data displayed on this track can be accessed through a hyperlinked PubMed identifier (PMID). This feature will enable the collation and display of ongoing proteomics data in the model plant Arabidopsis. In the initial release of the MASCP Gator the only posttranslational modification (PTM) displayed was phosphorylation. More recently we have endeavored to add and annotate an expanded array of PTMs that have been characterized in the literature and currently not readily accessible. The following have been recently added to the MASCP Gator as discrete tracks for visualization:

1. Ubiquitination, a covalent attachment of ubiquitin that typically occurs to lysine residues of proteins. These modifications can affect protein interactions and targeting as well as signal their degradation via the proteasome (Vierstra, 2009). The MASCP Gator currently displays 317 experimentally determined ubiquitination sites from 214 distinct Arabidopsis proteins defined from four studies (Maor et al., 2007; Saracco et al., 2009; Book et al., 2010; Kim et al., 2013). These modification sites are labeled with UBQ on a dedicated track.
2. Protein glycosylation represents one of the most complex PTMs in protein biochemistry and affects protein folding, stability, function and targeting (Gomord et al., 2010). In plants, few studies have attempted to extensively characterize this modification. Recently, a large-scale analysis of *N*-linked glycosylation was undertaken with nearly 2200 sites identified in Arabidopsis (Zielinska et al., 2012). These data have a dedicated glycosylation track labeled with CHO within the interface.
3. Methionine oxidation is generally regarded as a non-biological modification of proteins that occurs during sample preparation. While the oxidation of methionine residues as a consequence of cellular oxidative damage would provide a biological source of this PTM, its role in cellular signaling is also being explored (Hardin et al., 2009). A recent proteomic profile of methionine oxidation after the addition of a cGMP analog resulted in the identification of nearly 500 induced sites from nearly 400 proteins (Marondedze et al., 2013). These modifications are contained on a dedicated track and each site labeled MOX.
4. The N-terminal processing of targeting signals or presequences is a common feature of proteins imported into the mitochondrion or plastid. This processed polypeptide represents the mature form of the protein. A number of studies have characterized the N-terminal of these mature organelle proteins using proteomic approaches (Zybailov et al., 2008; Huang et al., 2009); while various studies have also defined these mature N-terminal sites by Edman degradation (Prime et al., 2000; Kruft et al., 2001; Eubel et al., 2003; Millar et al., 2004). Collectively, over 130 proteins have had their N-terminal presequence defined. The first amino acid is labeled with “MAT” in the MASCP Gator to indicate the start of the mature protein.
5. RNA editing is a process that results in posttranscriptional changes to mRNA that can often lead to changes to the resultant protein sequence. RNA editing in plants results in the conversion of cytidine to uridine and has thus been characterized in transcripts from mitochondria and plastids (Takenaka et al., 2008). A total of 478 RNA editing sites from fifty plastid and mitochondrial encoded genes that result in changes to the amino acid sequence are now available (Giege and Brennicke, 1999; Chateigner-Boutin and Small, 2007; Bentolila et al., 2008; Zehrmann et al., 2008). These changes are displayed with the substituted residue in a dedicated track RNA editing track.
6. *S*-Nitrosylation is the reversible covalent attachment of nitric oxide to the thiol groups of cysteine residues. Nitric oxide appears to be an important signaling molecule in plant biology with an involvement in processes such as seed germination and floral senescence while also having a role in abiotic and biotic stresses. Its role in signaling appears to be directly linked to the modification of target proteins. Few studies have attempted to characterize the actual modified cysteine residues, and currently only about 60 sites have been mapped in Arabidopsis (Dixon et al., 2005; Palmieri et al., 2010; Fares et al., 2011). These modifications are labeled with SNO in the interface on a dedicated track.

In addition to these newly added proteomic-based resources, data from the Arabidopsis 1001 Genomes Project have also been incorporated (Weigel and Mott, 2009). These data represent non-synonymous single nucleotide polymorphisms (nsSNPs) resulting from the re-sequencing of Arabidopsis accessions (Joshi et al., 2012). The data is presented as amino acid substitutions which are scored according to their frequency of observation in each accession, relative to *Arabidopsis thaliana* (Col-0).

## Enhancements to the MASCP gator

A number of enhancements have been made to both the user interface and to server side components. Mobile devices are taking an increasing share of the personal computing market, and as such it is useful to develop web applications so that they can work well on these devices. Further, as this is the direction that the computing space is heading, it is prudent to build applications so that they support this computing paradigm natively. Two challenges need to be addressed when retro-fitting an application to support this usage model; firstly, the computational components need to be optimized so that the application is responsive on a lower powered CPU as found on these devices. The second challenge faced is that the user interface needs to be updated so that it does not rely upon a mouse for interaction. The MASCP Gator has been updated to support both these challenges and as a result is more responsive on both mobile and desktop devices. To further improve the utility and speed of the MASCP Gator a server-side component has been established. Previously, it was necessary to set up a proxy server to allow access to certain sites that had not yet fully implemented the data interchange interface. In addition, software was required to allow for hosting of data sets that were retrieved from the literature. These two pieces of functionality were consolidated to enable periodic crawling and the update of data sources from the data providers.

The user interface has now been enriched to allow for the reloading of individual data types. Since the portal employs client side caching, data from a prior visit may be outdated. The Data sources component of the display now provides a timestamp to indicate the age of the displayed data. If necessary, individual data sources can be reloaded by clicking the circular arrow associated with each data type. A pop-up window has been added to the peptide tracks that displays the peptide sequence when the user hovers the mouse over an experimentally identified peptide on a track. This peptide sequence information can be copied using the mouse. A peptide search feature has also been added to the interface to enable a user to locate a peptide sequence in the currently displayed protein. If found, the peptide will be highlighted through the data tracks to provide context. The data tracks can now be manipulated and removed from the display. An “Edit” button on the track navigation section enables data track manipulation. Once activated, clicking the “X” adjacent to a given track will remove it from view. The track can be retrieved by refreshing the web page. The order of tracks can be manipulated by dragging and moving them while in edit mode. These features enable a better user experience with the resource.

## Future development

The incorporation and aggregation of new proteomic resources will be a primary driver of the MASCP Gator's future development. These data will likely comprise supplementary material from published studies and the integration of new independent databases. While minor modifications to the user interface will occur, the ongoing collection and visual display of proteomics data is the primary function of this resource. With the ongoing development of the Arabidopsis Information Portal (International Arabidopsis Informatics, 2012) by the Arabidopsis Informatics Consortium it is likely that much of the functionality present in the MASCP Gator will migrate to this new portal. The development of services by the Arabidopsis proteomics community that have enabled the construction of the MASCP Gator will provide a seamless transition to the Arabidopsis Information Portal in the near future.

## Author contributions

The manuscript was devised and written by Gregory W. Mann, Hiren J. Joshi, and Joshua L. Heazlewood. Data was collected and figures were created by Gregory W. Mann. Data updates for this implementation of the MASCP Gator were curated and added by Gregory W. Mann and Paul C. Calley. The core functionality of the MASCP Gator was developed by Hiren J. Joshi.

### Conflict of interest statement

The authors declare that the research was conducted in the absence of any commercial or financial relationships that could be construed as a potential conflict of interest.

## Supplementary material

Figure S1 **Histogram of protein coverage (%) for the 24,811 proteins with peptide data currently displayed in the MASCP Gator.** Greater than 50% of identified proteins have over 20% protein coverage.
