# Supplementary figures and images for: MASCP gator: an overview of the Arabidopsis proteomic aggregation portal
Source: Front Plant Sci. 2013 Oct 23;4:411. doi: 10.3389/fpls.2013.00411 (PMC3806167; doi:10.3389/fpls.2013.00411)

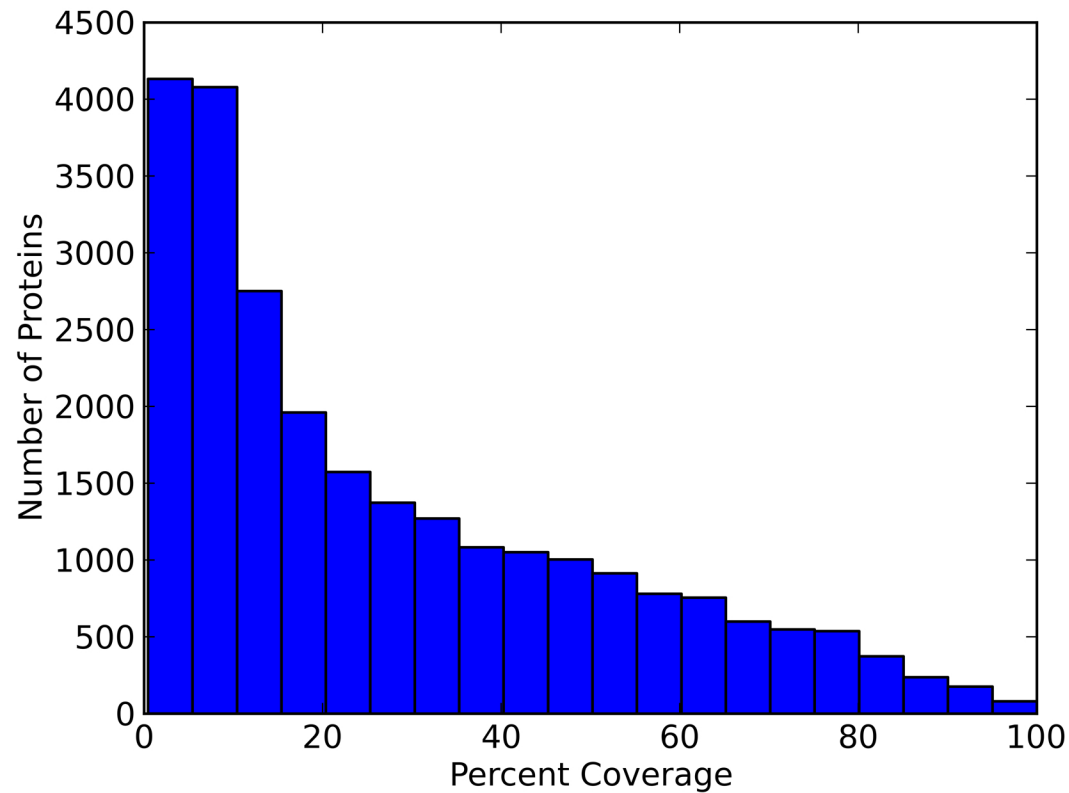

Figure S1

Supplement: Figure S1 — Histogram of protein coverage (%) for the 24,811 proteins with peptide data currently displayed in the MASCP Gator. Greater than 50% of identified proteins have over 20% protein coverage. [file Presentation1.PDF]
